# Integrated Analysis of mRNA-Seq and MiRNA-Seq Reveals the Molecular Mechanism of the Intestinal Immune Response in *Marsupenaeus japonicus* Under Decapod Iridescent Virus 1 Infection

**DOI:** 10.3389/fimmu.2021.807093

**Published:** 2022-01-18

**Authors:** Zihao He, Yunqi Zhong, Danqing Hou, Xianye Hu, Zhibin Fu, Luyao Liu, Shuang Zhang, Chengbo Sun

**Affiliations:** ^1^ College of Fisheries, Guangdong Ocean University, Zhanjiang, China; ^2^ Aquatic Animals Precision Nutrition and High Efficiency Feed Engineering Research Center of Guangdong Province, Zhanjiang, China; ^3^ Guangdong Provincial Laboratory of Southern Marine Science and Engineering, Zhanjiang, China; ^4^ Guangdong Provincial Key Laboratory of Pathogenic Biology and Epidemiology for Aquatic Economic Animals, Zhanjiang, China

**Keywords:** decapod iridescent virus 1, *Marsupenaeus japonicus*, miRNA, mRNA, intestinal immune response

## Abstract

The intestine is not only an important digestive organ but also an important immune organ for shrimp; it plays a key role in maintaining homeostasis. Decapod iridescent virus 1 (DIV1) is a new type of shrimp-lethal virus that has received extensive attention in recent years. To date, most studies of the shrimp intestinal immune response under viral infections have relied on single omics analyses; there is a lack of systematic multi-omics research. In the current study, intestinal mRNA-seq and microRNA (miRNA)-seq analyses of *Marsupenaeus japonicus* under DIV1 infection were performed. A total of 1,976 differentially expressed genes (DEGs) and 32 differentially expressed miRNAs (DEMs) were identified. Among them, 21 DEMs were negatively correlated with 194 DEGs from a total of 223 correlations. Functional annotation analysis revealed that *M. japonicus* can regulate glycosaminoglycan biosynthesis (chondroitin sulfate, dermatan sulfate, and keratan sulfate), vitamin metabolism (retinol metabolism and ascorbate and aldarate metabolism), immune pathway activation (Toll and IMD signaling pathways, Wnt signaling pathway, IL-17 signaling pathway, and Hippo signaling pathway), immunity enzyme activity promotion (triose-phosphate isomerase), antimicrobial peptide (AMP) expression, reactive oxygen species (ROS) production, and cell apoptosis through miRNAs to participate in the host’s antiviral immune response, while DIV1 can influence Warburg effect-related pathways (pyruvate metabolism, glycolysis/gluconeogenesis, and citrate cycle), glycosphingolipid biosynthesis-related pathways (glycosphingolipid biosynthesis—globo and isoglobo series and glycosphingolipid biosynthesis—lacto and neolacto series), and the tight junction and adhesion junction of the intestinal mucosal epithelium through the host’s miRNAs and mRNA to promote its own invasion and replication. These results indicate that intestinal miRNAs play important roles in the shrimp immune response against DIV1 infection. This study provides a basis for further study of the shrimp intestinal antiviral immune response and for the formulation of effective new strategies for the prevention and treatment of DIV1 infection.

## 1 Introduction

According to the Food and Agriculture Organization (FAO) of the United Nations, the total world production of farmed shrimp exceeded 5,700 thousand tonnes in 2018 (1). The shrimp farming industry has become an important pillar industry within the aquaculture industry. However, as the production and density of shrimp aquaculture continue to expand, various diseases, especially viral diseases, occur more frequently. At present, there are more than 20 kinds of shrimp viral pathogens that have been discovered globally, including the common white spot syndrome virus (WSSV) (2), infectious hypodermal and hematopoietic necrosis virus (IHHNV) (3), yellow head virus (YHV) (4), and Taura syndrome virus (TSV) (5). Moreover, new shrimp viruses continue to be discovered. The diseases caused by these newly discovered viruses not only spread rapidly around the world but also cause serious economic losses to coastal farmers.

The intestine represents the largest compartment of the immune system. It is continually exposed to antigens and immunomodulatory agents from the diet and the commensal microbiota, and it is the port of entry for many clinically important pathogens. Intestinal immune processes are also increasingly implicated in controlling disease development elsewhere in the body.

Decapod iridescent virus 1 (DIV1) is a newly discovered enveloped icosahedral virus that was first described as *Cherax quadricarinatus* iridovirus (CQIV CN01) infectious to *C. quadricarinatus* (6) or shrimp hemocyte iridescent virus (SHIV 20141215) infectious to *Litopenaeus vannamei* (7). Because the genomic similarity between these two original isolations was 99%, the Executive Committee of the International Committee on Taxonomy of Viruses (ICTV) denoted CQIV CN01 and SHIV 20141215 as two virus isolates of DIV1 (8, 9). At present, research on DIV1 is still in the preliminary stage. Through detection methods such as nest PCR, TaqMan probe-based real-time PCR, and qLAMP, researchers have found that DIV1 has a wide range of hosts, including several economically cultured species such as *L. vannamei*, *Fenneropenaeus merguiensis*, *C. quadricarinatus*, *Exopalaemon carinicauda*, *Macrobrachium rosenbergii*, *Penaeus monodon*, and *Marsupenaeus japonicus* (6, 10–15). In addition, DIV1 also has a very high fatality rate, which brings huge economic losses to shrimp farmers (16, 17). A recent study reported that DIV1 was detected in wild *P. monodon* captured from the north-eastern Indian Ocean (18), indicating that this virus has spread outside of China. This means that DIV1 may have spread worldwide through wild shrimp, bringing new threats to the sustainable development of the global shrimp farming industry.

As an arthropod species, shrimp lack adaptive immunity and avoid diseases by relying exclusively on the innate immune system, which is the first line of defense against pathogenic infections that triggers diverse humoral and cellular activities *via* signal transduction pathways (19). Therefore, improvement of self-immunity is the fundamental measure of disease control in shrimp, and the investigation of immune defense mechanisms for disease control is of great significance (20, 21). In order to identify approaches to control DIV1, researchers have studied the immune responses of different species of shrimp infected with DIV1 through transcriptome analyses. The results showed that shrimp can resist DIV1 infection by regulating the expression of triose-phosphate isomerase (TPI), caspases, C-type lectins, heat shock protein 70 (HSP70), crustins, and other immune genes (11–14, 22). These studies focused on the immune response to DIV1 in shrimp hemocytes and the hepatopancreas. However, there are no published reports on the immune response in the intestine. The intestine, an important digestive and immune organ in shrimp, can resist foreign pathogens through mechanical, immune, and biological barriers (23–25). Many studies have demonstrated that the addition of dietary supplements such as probiotics, poly-β-hydroxybutyrate, and peptides can effectively improve the activity of shrimp intestinal immune enzymes and the expression of immune genes, thereby enhancing the host’s antiviral and anti-stress activities (26–29). Kuruma shrimp *M. japonicus* is one of the most important farmed species in the shrimp farming industry. Our previous study demonstrated that *M. japonicus* is a susceptible host to DIV1 and showed that shrimp hemocytes play an important role in resisting DIV1 infection (12) . In addition, one of the distinct clinical signs of shrimp after DIV1 infection was empty guts (15). Thus, an in-depth understanding of the intestinal immune response of shrimp during DIV1 infection will help with the development of new strategies for the effective control of shrimp viral diseases.

MicroRNAs (miRNAs) are a class of small noncoding RNAs composed of about 22 nucleotides (nt). This class of RNA is widely present in organisms and participates in a variety of biological processes such as growth and development, metabolism, apoptosis, signal transduction, and immune defense by inhibiting mRNA translation or degrading mRNA (30, 31). With the widespread application of high-throughput sequencing and the improvement in shrimp transcriptome data, more and more shrimp miRNAs have been identified and have been found to have antiviral effects. On the one hand, miRNAs can improve the antiviral ability of shrimp by participating in phagocytosis, apoptosis, the prophenoloxidase cascade, and antimicrobial peptide (AMP) production (32, 33). On the other hand, miRNA can exert antiviral effects by inhibiting the expression of early viral genes or triggering the degradation of viral genes (34, 35). Although several studies of miRNA have been performed in shrimp, the role of miRNAs in the intestinal antiviral immune response of shrimp is still unclear.

In this study, RNA sequencing was first used to separately study the expression profiles of miRNA and mRNA in the intestine of *M. japonicus* during DIV1 infection. Then, an integrated analysis of mRNA-seq and miRNA-seq was performed. The results contribute to an understanding of the molecular mechanism of shrimp intestinal antiviral infection and viral immune evasion and provide a theoretical basis for virus control technology.

## 2 Materials and Methods

### 2.1 Shrimp Culture and Decapod Iridescent Virus 1 Challenge

Healthy *M. japonicus* (body weight 10.5 ± 1.6 g) were initially acclimatized for 1 week in 0.3-m^3^ tanks with aerated and filtered seawater at the East Island Marine Biological Research Base, Guangdong Ocean University in Zhanjiang, Guangdong, China. The shrimp were randomly sampled and tested by PCR to ensure that they were free from WSSV, IHHNV, and DIV1. The holding seawater conditions were as follows: salinity at ~30‰, pH at ~7.5, and temperature at ~28°C. The shrimp were fed three times daily at a rate of 5% of body weight, and nearly 90% of the seawater was changed once per day. After 7 days, the shrimp were randomly divided into the DIV1-infected group and negative control group, with each group containing 30 *M. japonicus*.

In the DIV1 challenge experiment, each *M. japonicus* from the DIV1-infected group was intramuscularly injected with 50 μl of DIV1 inoculum (3.95 × 10^9^ copies/μg DNA, identified by qPCR), while each *M. japonicus* from the negative control group was intramuscularly injected with 50 μl of phosphate-buffered saline (PBS; pH 7.4). The methods for virus extraction and quantification have been described in detail previously (He et al., 2021). Twenty-four hours post-injection (hpi), the intestines of three individuals in the same group were collected and combined as one sample under aseptic conditions. The samples were immediately frozen in liquid nitrogen and then stored at −80°C until RNA extraction. The study protocol was approved by the ethics review board of the Institutional Animal Care and Use Committee of Guangdong Ocean University.

### 2.2 RNA Extraction

Three samples from the DIV1-infected group and three samples from the negative control group were randomly selected. The intestines of these shrimp were ground into a powder with liquid nitrogen before total RNA extraction. Total RNA was extracted separately from the intestines using TRIzol (Invitrogen, Carlsbad, CA, USA), according to the manufacturer’s protocol. The concentration of total RNA was determined using a NanoDrop (Thermo Fisher Scientific, MA, USA), and the integrity was examined by electrophoresis on 2% agarose gel.

### 2.3 mRNA Sequencing and Data Analysis

For the construction of the six cDNA libraries, total RNA was extracted from each sample using a TranZol Up Plus RNA Kit (Transgen, Beijing, China), according to the manufacturer’s protocol, and the RNA concentration was determined with an Agilent 2100 bioanalyzer. Oligo (dt)-attached magnetic beads were used to purify the mRNA. Purified RNA was fragmented into small pieces with fragment buffer at an appropriate temperature. With the use mRNA as a template, the first-strand cDNA was generated in First Strand Master Mix by PCR, and the second-strand cDNA was also generated. After purification and elution with EB buffer, cDNA end-repair and adenylation at the 3′ end were performed, and then poly(A) was added, and the sequencing adaptor was connected. The libraries were validated on an Agilent 2100 bioanalyzer for quality control. Finally, mRNA sequencing was performed by BGI (Shenzhen, China) using Illumina Genome Analyzer technology.

The raw reads were filtered to remove adaptor and low-quality sequences using SOAPnuke software (v1.4.0) (36). Then, the clean reads were assembled using Trinity software (v2.0.6) for *de novo* transcriptome assembly without a reference genome (37). Based on the sequence similarity and length, TGICL (v2.1) was used to remove redundant sequences and generate unigenes (38). The completeness of the assembly was assessed using BUSCO (v3.0.2) with the BUSCO arthropod dataset (39). Transcript abundance was estimated using RSEM (v1.2.8) (40). The assembled unigenes were annotated with six functional databases, including Kyoto Encyclopedia of Genes and Genomes (KEGG; https://www.genome.jp/kegg), Gene Ontology (GO; https://geneontology.org), Clusters of Orthologous Groups for Eukaryotic Complete Genomes (KOG; https://www.ncbi.nlm.nih.gov/COG/), Non-Redundant Protein Sequence Database (Nr; https://ftp.ncbi.nlm.nih.gov/blast/db), Protein Families Database (Pfam; http://pfam.xfam.org), and the Swiss-Prot Protein Sequence Database (SwissProt; http://www.uniprot.org/). Differentially expressed genes (DEGs) in the two groups were detected using the DESeq2 package (1.30.0) (41, 42). The *p*-value was adjusted using the *Q*-value (43), and genes with a *Q*-value < 0.05 and |log_2_(fold change)| > 1 by DESeq2 were considered to be DEGs. All DEGs were further analyzed using the GO and KEGG databases to identify significantly enriched GO terms and KEGG pathways.

### 2.4 Small RNA Sequencing and Data Analysis

Each of the six small RNA libraries was prepared with 1 μg of total RNA per sample. Total RNA was purified by electrophoretic separation on 15% urea-denaturing polyacrylamide gel electrophoresis (PAGE) gel, and small RNAs between 18 and 30 nt were excised and recovered. Next, the small RNAs were ligated to adenylated 3′ adapters and annealed to unique molecular identifiers (UMIs). Then, ligation of 5′ adapters was performed. The adapter-ligated small RNAs were subsequently transcribed into cDNA by SuperScript II Reverse Transcriptase (Invitrogen, USA) and amplified by PCR. The target PCR products were purified through 4% agarose gels and were prepared for Illumina sequencing. The libraries were also validated on an Agilent 2100 bioanalyzer for quality control. The qualified libraries were sequenced by BGI (Shenzhen, China) with Illumina Genome Analyzer technology.

After high-throughput small RNA sequencing was completed, the clean tags were obtained from the raw tags by filtering out low-quality tags, invalid adapter tags, poly(A) tags, short valid tags, and long valid tags. After filtering, the clean tags were mapped to the *L. vannamei* genome (NCBI Assembly GCA_003789085.1) in GenBank and other small RNA databases including miRbase (22.1, http://www.mirbase.org), siRNA, piRNA, and snoRNA with Bowtie2 (2.2.9) (44). Further, cmsearch (1.1.2) (45) was used for Rfam mapping (http://rfam.xfam.org) (46). The novel miRNA candidates were predicted using miRDeep2 (2.0.0.8, https://github.com/rajewsky-lab/mirdeep2) (47) based on the hairpin-like secondary structure pattern. In order to understand the changes in the expression levels of intestinal miRNAs in *M. japonicus* before and after infection with DIV1, the expression levels of known miRNAs and novel miRNAs were calculated by counting the absolute numbers of molecules using UMI (48). Then, a *Q*-value < 0.05 and |log_2_(fold change)| > 0.1 were set as the threshold for identification of differentially expressed miRNAs (DEMs).

The target genes of DEMs were predicted with RNAhybrid (49) and miRanda (50). Overlapping target genes were selected for further analysis. To identify the potential biological functions of the target genes and the main pathways targeted by the gene candidates, enrichment analysis of these predicted target genes was performed for the GO term and KEGG pathways.

### 2.5 Quantitative Real-Time PCR Validation of Differentially Expressed Genes and Differentially Expressed MiRNAs

To validate the gene expression profiles from the Illumina sequencing results, 15 mRNAs and 11 miRNAs were randomly selected to perform qRT-PCR. The primers for qRT-PCR were designed by Primer 5.0 software and are listed in **Table 1**. The housekeeping gene for the qRT-PCR analysis of DEGs was *Elongation factor-1 gene alpha* (*EF-1α*, LOC122259198) of *M. japonicus* and U6 for DEMs. qRT-PCR was performed using the methods described in detail previously (51, 52). For DEG validation, high-quality RNA was reverse-transcribed using 5× All-in-One RT Master Mix (Applied Biological Materials, Vancouver, Canada), according to the manufacturer’s protocol. Then, qRT-PCR was performed using a TB Green^®^ Premix Ex Taq™ Kit (TaKaRa, Japan); *EF-1α* of *M. japonicus* was used as the internal reference gene. qRT-PCR was performed with 20 μl of reaction mixture containing 10 μl of TB Green^®^ Premix Ex Taq™, 1 μl of each primer (10 μM), 1 μl of diluted cDNA template, and 7 μl of ultrapure water. The reaction cycle parameters were as follows: 95°C for 2 min, 40 cycles of 95°C for 5 s, and holding at 60°C for 30 s. The DEM results were validated by poly(A) tailing-based reverse transcription PCR (53) using a miRNA First Strand cDNA Synthesis Kit (Tailing Reaction) (Sangon Biotech, Shanghai, China). Then, qRT-PCR was performed using a miRNA qPCR Kit (SYBR Green Method) (Sangon Biotech, Shanghai, China); U6 was used as an internal control. qRT-PCR was performed with 20 μl of reaction mixture containing 10 μl of 2× miRNA qPCR Master Mix, 0.5 μl of forwarding primer (10 μM), 0.5 μl of Universal PCR Primer R (10 μM), 2 μl of diluted cDNA template, and 7 μl of RNase-free water. The reaction cycle parameters were as follows: 95°C for 30 s, 40 cycles of 95°C for 5 s, and holding at 60°C for 30 s. All reactions were performed in three technical replicates. The relative expression levels were calculated using the 2^−ΔΔCt^ method (54); log_2_ (fold change) was used to show the differential expression of mRNA or miRNA in the DIV1-infected group and negative control group.

**Table 1:** Details of the primer sequence used for qRT-PCR.

No.	Primer Names	Sequences (5′–3′)
	**mRNA-seq**	
1	Unigene15935_All-F	TCTGACCGCTGAGAACTTTG
2	Unigene15935_All-R	TCTACGAGCTAGAGCTGATGTG
3	CL1405.Contig4_All-F	TTCTGGCTGCTGTAAGAGTGA
4	CL1405.Contig4_All-R	TTGCTGGAGTGGTACGTGAT
5	CL3214.Contig2_All-F	CCCTGATTTCCCTTGCTTC
6	CL3214.Contig2_All-R	TCTTCCTCTTTGCCGTCCT
7	CL1030.Contig_All4-F	TTTGCCCGTGACTTTGTTC
8	CL1030.Contig4_All-R	CAGTTCGGATTTCCCCTCT
9	Unigene10378_All-F	AACATCATCCCTTGAACCCG
10	Unigene10378_All-R	CGCTGTCTTTTGCTTCGTTG
11	CL2943.Contig2_All-F	AGAATCCTATCCCCTCGGTA
12	CL2943.Contig2_All-R	TGCTCATACTTGTGATGTCGC
13	CL3345.Contig1_All-F	GCAATGTCGTGGATAAGCG
14	CL3345.Contig1_All-R	AAGCAGGGAGGCAATGAGT
15	CL2966.Contig1_All-F	CGCCCATTTCTTCGTTTC
16	CL2966.Contig1_All-R	TGTGCTGCCATGTCGTTAA
17	CL2784.Contig5_All-F	TCTACTTCGTCAAGGTGGGC
18	CL2784.Contig5_All-R	TCCGTCGTCATCCGTATCTC
19	CL709.Contig1_All-F	TTTGTCTTGCTGCCTTCCTA
20	CL709.Contig1_All-R	GAGTCCTGTGGTGGTTTTGAG
21	CL2520.Contig2_All-F	TCATCCACCGTTTTCTACCC
22	CL2520.Contig2_All-R	CTTGATTACATTGCCCTTGC
23	Unigene18060_All-F	CAGCAGAGTTTTCCCCACA
24	Unigene18060_All-R	TATTTCGAGACGGCAGAGC
25	CL3472.Contig2_All-F	GCTGAACCTGCTGGAATGA
26	CL3472.Contig2_All-R	TGGAGACTGCTGTCCCTTGT
27	CL1127.Contig3_All-F	CCAGGCTACAAAGTGGAGTG
28	CL1127.Contig3_All-R	GTCTTGGCAACATTGGTGAA
29	CL2402.Contig3_All-F	CACCAACGGAAAGACCTACTC
30	CL2402.Contig3_All-R	AGACCGCTGCCTTGAAATA
31	MjEF1-α-F	GGAACTGGAGGCAGGACC
32	MjEF1-α-R	AGCCACCGTTTGCTTCAT
	**MiRNA-seq**	
33	miR-193-3p_6-F	TACTGGCCTGCTAAGTCCCAA
34	miR-263b-F	CTTGGCACTGGAAGAATTCACAGA
35	miR-6493-5p-F	ACGTCCGGCAGGTTTTACC
36	novel_mir86-F	TATATATATATAGCGCGCGGGCGG
37	miR-263a-5p_1	AATGGCACTGGAAGAATTCACGG
38	novel_mir56	TATATATATAGGCGGGGGCTGGC
39	miR-750_3-F	CGCAGATCTAACTCTTCCAGCTCA
40	novel_mir63-F	TATATATACGCGTGGGGGATGACG
41	novel_mir82-F	TATATAGCGCGGCCGGATG
42	novel_mir40-F	ACGCCTTTGGTTTTACGGTCTTC
43	novel_mir58	TCTCTGCGGCTCTTGGC
44	U6-F	CGCAAGGATGACACGCAAATT
45	Reverse Primer	Universal PCR Primer R (Sangon Biotech)

### 2.6 Co-Analysis of Small RNA Sequencing and mRNA Sequencing

In order to further study the interaction between DEMs and DEGs in the intestine of *M. japonicus* under DIV1 infection, the statistical package R was used to calculate Pearson’s correlation coefficients of DEMs/DEGs based on the expression levels in small RNA sequencing and mRNA sequencing. A Pearson’s correlation coefficient > 0.6 and *p* < 0.05 were considered to be a strong correlation. Considering the negative regulatory relationship between miRNA and target genes, enrichment analysis of GO terms and KEGG pathways was performed on miRNA–mRNA pairs with a Pearson’s correlation coefficient <−0.6 and *p* < 0.05.

### 2.7 Statistical Analysis

The data are expressed as the mean ± SD. Data normality was checked by the Shapiro–Wilk test. One-way ANOVA and multiple comparison Tukey’s tests were used for between-group comparisons. All statistical analyses were performed using SPSS 19.0 (SPSS Inc., Chicago, IL, USA). A probability level of 0.05 was used to indicate statistical significance (*p* < 0.05).

## 3 Result

### 3.1 mRNA Expression Profiling

#### 3.1.1 mRNA Sequencing and *De Novo* Assembly

**Figure 1:**
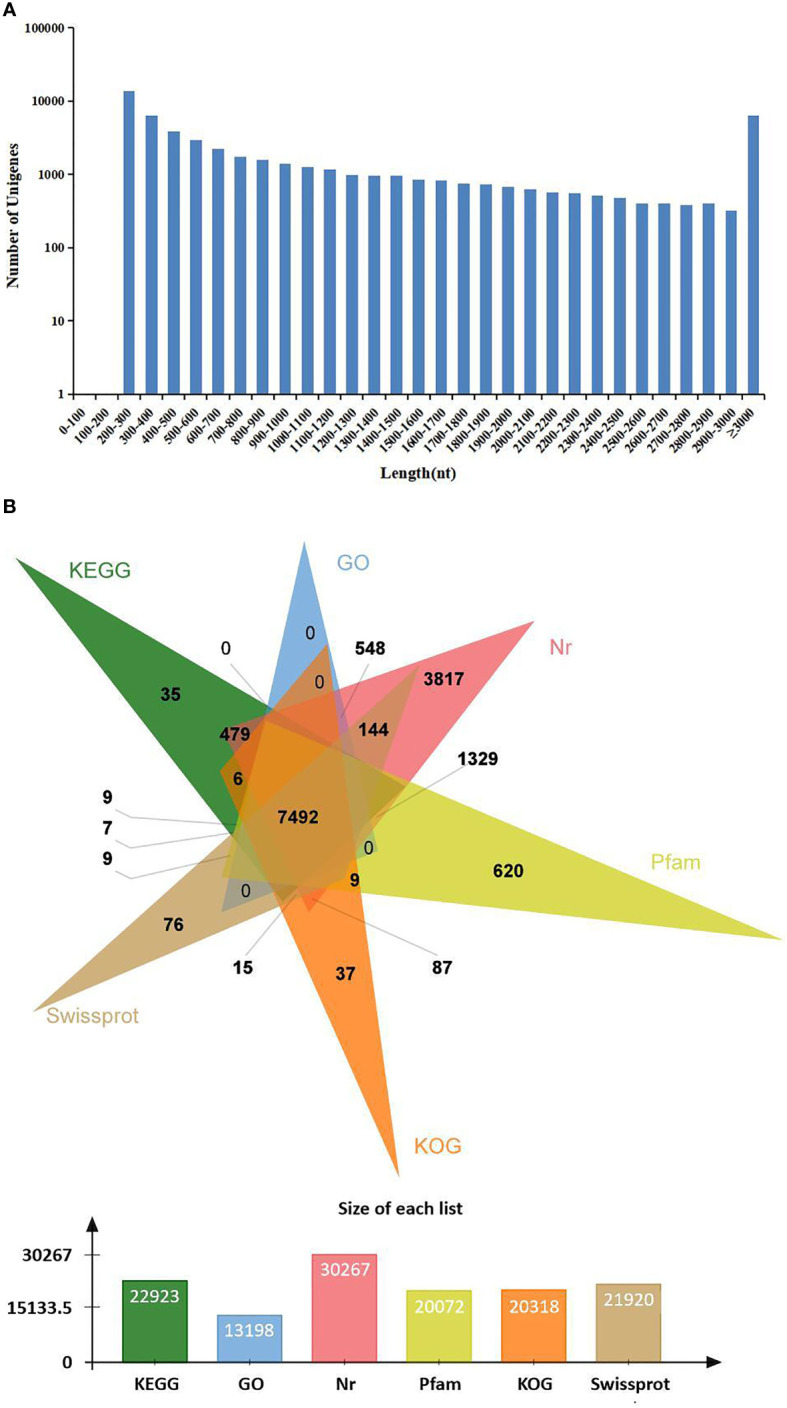
Length distribution and annotation of transcriptome unigenes. **(A)** Length distribution of transcriptome unigenes. The x-axis indicates the length of unigenes, and the y-axis indicates the number of unigenes. **(B)** Annotation of transcriptome unigenes. The Venn diagram shows the annotation of unigenes from the *Marsupenaeus japonicus* transcriptome in 6 databases, including the KEGG, GO, KOG, Nr, Pfam, and SwissProt databases. KEGG, Kyoto Encyclopedia of Genes and Genomes; GO, Gene Ontology.

In order to identify mRNA expression profiles in the *M. japonicus* intestine under DIV1 infection, six cDNA libraries representing DIV1-infected and non-infected *M. japonicus* were constructed with total RNA and were then subjected to Illumina deep sequencing. After quality filtering, a total of 128,936,186 and 128,535,448 clean reads representing a total of 19.34 and 19.28 Gb nucleotides were generated for the negative control and DIV1-infected groups, respectively. The guanine–cytosine (GC) content of clean reads was 43.06% in the negative control group and 42.56% in the DIV1-infected group (**Table 2**). After the redundant assembled contigs were removed, a total of 54,107 unigenes were retained. The size and length distribution of the negative control group and DIV1-infected group unigenes are shown in **Figure 1A**. Most of the unigenes (13,726, 25.37%) were 200–300 nt in length, followed by 300–400 nt (6,299, 11.64%); 6,363 unigenes (11.76%) were ≥3000 nt in length.

**Table 2:** Summary of mRNA-seq data.

Samples	Raw Reads Number	Clean Reads Number	Clean Bases (Gb)	Q20 (%)	Q30 (%)	Clean Reads Ratio (%)	GC (%)
DIV1-1	43,821,050	42,839,474	6.43	96.62	91.58	97.76	43.06
DIV1-2	43,821,050	42,765,910	6.41	96.85	92.09	97.59	42.65
DIV1-3	43,821,050	42,930,064	6.44	97.00	92.40	97.97	41.98
Control-1	43,821,050	43,144,600	6.47	96.60	91.45	98.46	42.92
Control-2	43,821,050	42,908,058	6.44	96.68	91.75	97.92	43.10
Control-3	43,821,050	42,883,528	6.43	96.81	92.02	97.86	43.15
Total	306,747,350	300,355,162	45.05	96.79	91.95	97.89	42.98

GC, guanine–cytosine.

Sequencing reads were deposited into the Sequence Read Archive (SRA) of the National Center for Biotechnology Information (NCBI) and are available with the accession number PRJNA720250 (https://www.ncbi.nlm.nih.gov/sra).

#### 3.1.2 Annotation of the Assembled Sequences

**Figure 2:**
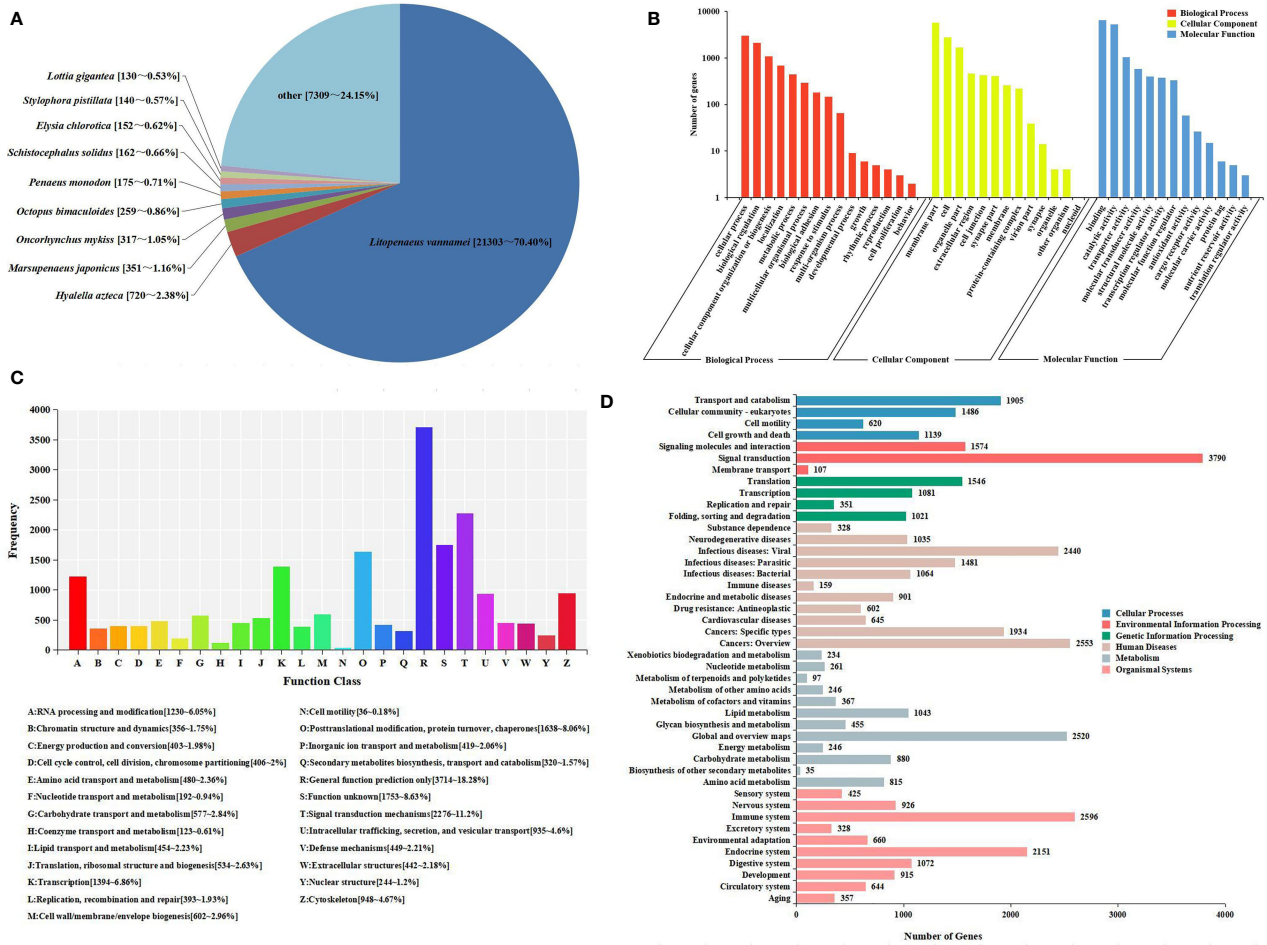
Sequence analysis and functional annotation of assembled unigenes identified from the *Marsupenaeus japonicus* intestine under DIV1 infection. **(A)** Species distribution of the BLASTx matches of the transcriptome unigenes. This figure shows the species distribution of unigene BLASTx matches against the Nr protein database and the proportions for each species. **(B)** GO terms (level 2) and annotation of the integrated transcriptome assembly. The x-axis indicates 3 GO categories with 41 subcategories, and the y-axis indicates the number of unigenes. **(C)** KOG function classification of unigenes. Each bar represents the number of unigenes classified into each of the 25 KOG functional categories. The x-axis represents the functional categories, and the y-axis represents the frequency. **(D)** KEGG biological pathway classification histograms for annotated unigenes. Each bar represents the number of unigenes classified into different biological processes. The x-axis shows the number of the matched unigenes, and the y-axis shows the pathways from the KEGG classification. DIV1, decapod iridescent virus 1; GO, Gene Ontology; KEGG, Kyoto Encyclopedia of Genes and Genomes.

All unigenes were searched against the KEGG, GO, KOG, Nr, Pfam, and SwissProt databases, returning 22,923 (42.37%), 13,198 (24.39%), 20,318 (37.55%), 30,267 (55.94%), 20,072 (37.10%), and 21,910 (40.49%) matches, respectively (**Figure 1B**). The species distribution of the most significant hits in the Nr database was examined to investigate the sequence conservation of *M. japonicus* compared with other species (**Figure 2A**). Over 75% of the total unigenes matched with sequences from five top-hit species—*L. vannamei* (70.40%), *Hyalella azteca* (2.38%), *M. japonicus* (1.16%), *Oncorhynchus mykiss* (1.05%), and *Octopus bimaculoides* (0.86%)—all of which are aquatic organisms (**Figure 2A**). Through GO annotation, 13,198 unigenes were aligned to 41 GO subcategories (level 2) within three overarching categories: biological process (8,048 unigenes, 15 subcategories), cellular component (12,031 unigenes, 13 subcategories), and molecular function (14,622 unigenes, 13 subcategories) (**Figure 2B**). In the biological process category, most unigenes were involved in “cellular process” and “biological regulation.” In the cellular component category, “membrane part” and “cell” were the most represented. For the molecular function category, “binding” and “catalytic activity” were the dominant groups. The KOG database was then used to further explore orthologs of the assembled unigenes; 20,318 unigenes were successfully annotated with 25 specific protein function definitions or orthologous categories (**Figure 2C**). Among these protein function categories, the largest three were “General function prediction only” (3,714 unigenes, 18.28%), “Signal transduction mechanisms” (2,276, 11.2%), and “Function unknown” (1,753, 8.63%). To identify the biological processes of the annotated unigenes, 22,923 unigenes were annotated using the KEGG database and assigned to different pathways in six major groups of KEGG pathways, including Cellular Processes, Environmental Information Processing, Genetic Information Processing, Human Diseases, and Metabolism and Organismal Systems. These annotated unigenes were further divided into 43 level 2 subcategories, except for “Global and overview maps,” which contained no pictorial information. The largest subcategory group was “Signal transduction” (3,790 unigenes), followed by “Immune system” (2,596 unigenes), “Cancers: Overview” (2,553 unigenes), and “Infectious diseases: Viral” (2,440 unigenes) (**Figure 2D**).

#### 3.1.3 Identification and Functional Characterization of Differentially Expressed Genes

**Figure 3:**
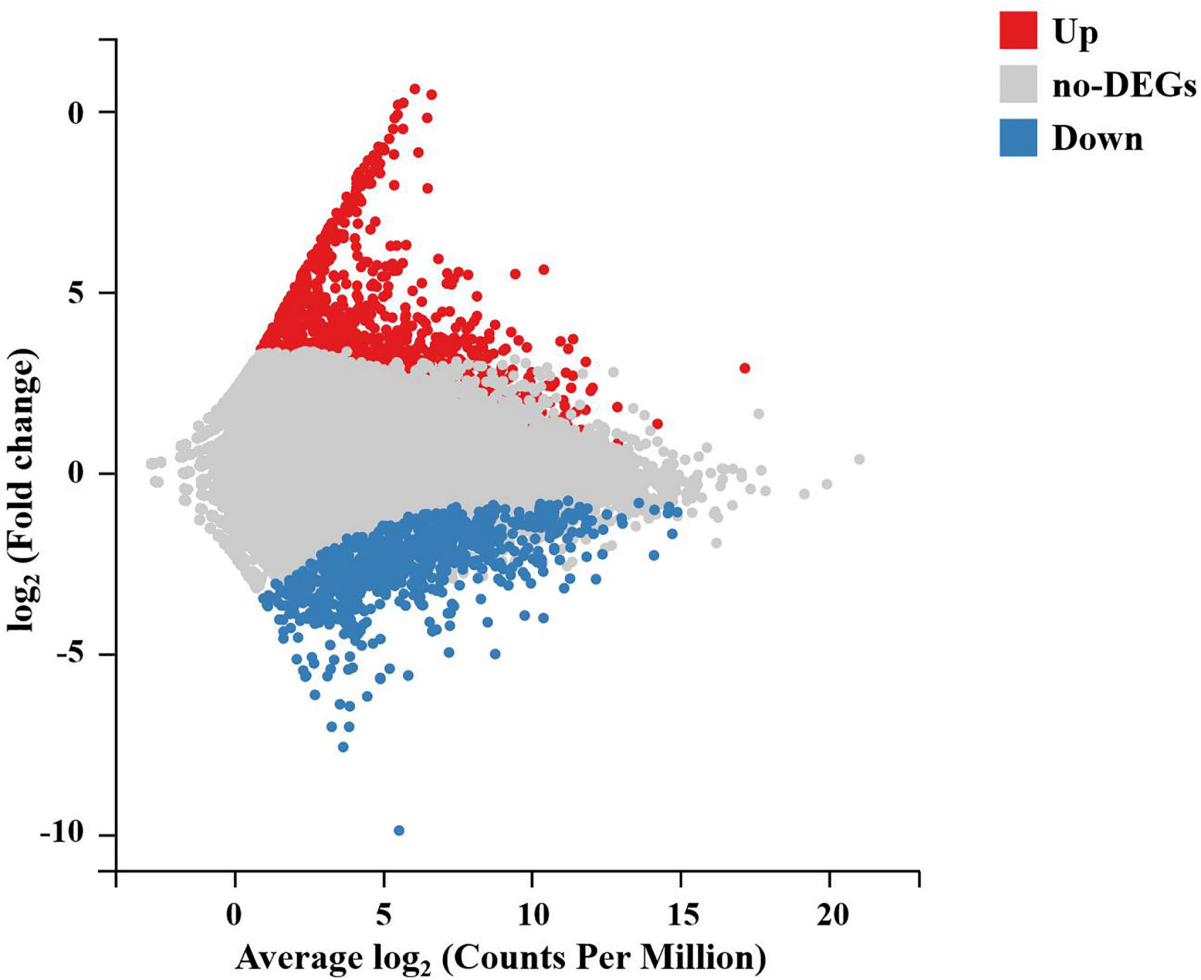
M-versus-A plot (MA plot) between DEGs of the DIV1-infected and negative control *Marsupenaeus japonicus* intestines. The x-axis indicates the average expression level, and the y-axis indicates the fold change. Red dots represent the significantly upregulated DEGs, while blue dots represent the significantly downregulated DEGs (*Q*-value < 0.05 and |log_2_(fold change)| > 1). Gray dots represent the DEGs that were not significantly different. DEGs, differentially expressed genes; DIV1, decapod iridescent virus 1.

To further analyze and characterize the DEGs, a total of 1,976 DEGs were screened, including 1,234 upregulated genes and 742 downregulated genes, with a *Q*-value < 0.05 and |log_2_(fold change)| > 1 as the cutoff (**Figure 3**). To further examine the functions of these genes and their related biological processes, GO term and KEGG pathway enrichment analyses were performed for the DEGs.

**Figure 4:**
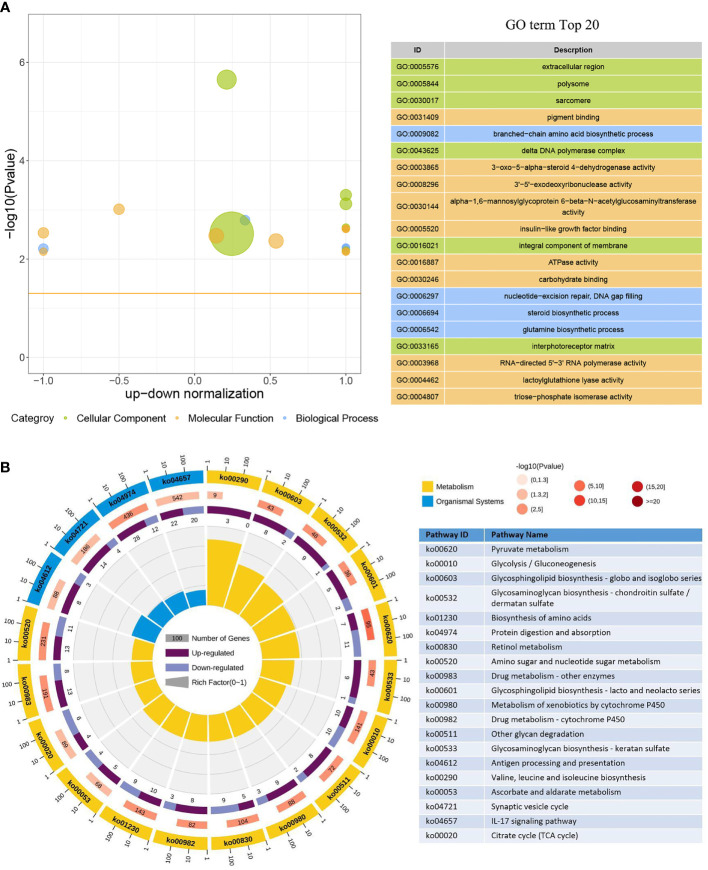
GO term and KEGG pathway enrichment analyses of DEGs. **(A)** GO bubble plot showing a summary of the top 20 enriched GO terms (level 3). Different colors represent different GO categories, including biological process, cellular component, and molecular function. The x-axis indicates up–down normalization, and the y-axis indicates −log_10_(*p*-value). **(B)** The first lap indicates the top 20 KEGG pathways (level 3), and the number of genes corresponds to the outer lap. The second lap indicates the number of genes in the genome background and the *Q*-values for the enrichment of the DEGs for the specified biological process. The third lap indicates the ratio of the upregulated genes (deep purple) and downregulated genes (light purple). The fourth lap indicates the enrichment factor of each KEGG term. GO, Gene Ontology; KEGG, Kyoto Encyclopedia of Genes and Genomes; DEGs, differentially expressed genes.

In the GO enrichment analysis, 386 upregulated genes and 246 downregulated genes expressed in the DIV1-infected group were divided into three categories with 30 subcategories (level 2): biological progress (10 subcategories), cellular component (10 subcategories), and molecular function (10 subcategories). The top 20 GO terms (level 3) influenced by DIV1 infection are shown in **Figure 4A**. Most of the corresponding DEGs were enriched in integral components of the membrane (188 upregulated genes and 114 downregulated genes) in the cellular component category. It is worth noting that the GO term “triose-phosphate isomerase activity” (2 upregulated genes), which may be related to DIV1 infection, was also significantly enriched in the GO enrichment analysis (13) .

Moreover, 473 upregulated genes and 318 downregulated genes were annotated into 238 pathways (level 3) in the KEGG pathway enrichment analysis. The top 20 KEGG pathways influenced by DIV1 infection were divided into two major groups, i.e., Metabolism and Organismal System (**Figure 4B**). Most of the DEGs were enriched in the IL-17 signaling pathway (22 upregulated genes and 20 downregulated genes), followed by Protein digestion and absorption (28 upregulated genes and 12 downregulated genes). Two pathways related to vitamin metabolism were significantly enriched under DIV1 infection, i.e., Retinol metabolism (5 upregulated genes and 9 downregulated genes) and Ascorbate and aldarate metabolism (5 upregulated genes and 4 downregulated genes); moreover, several metabolism pathways considered to be the hallmark pathways of the Warburg effect in vertebrates were significantly enriched, including Pyruvate metabolism (7 upregulated genes and 11 downregulated genes), Glycolysis/Gluconeogenesis (10 upregulated genes and 10 downregulated genes), and Citrate cycle (tricarboxylic acid (TCA) cycle, 4 upregulated genes and 6 downregulated genes). It is worth noting that pathways related to glycosphingolipid biosynthesis and glycosaminoglycan biosynthesis were also significantly enriched, including Glycosphingolipid biosynthesis—globo and isoglobo series (8 upregulated genes and 2 downregulated genes), Glycosphingolipid biosynthesis—lacto and neolacto series (5 upregulated genes and 2 downregulated genes), Glycosaminoglycan biosynthesis—chondroitin sulfate/dermatan sulfate (9 upregulated genes and 1 downregulated gene), and Glycosaminoglycan biosynthesis—keratan sulfate (6 upregulated genes and 1 downregulated gene).

### 3.2 MiRNA Expression Profiling

#### 3.2.1 MiRNA Sequencing and Annotation of the Clean Tags

**Figure 5:**
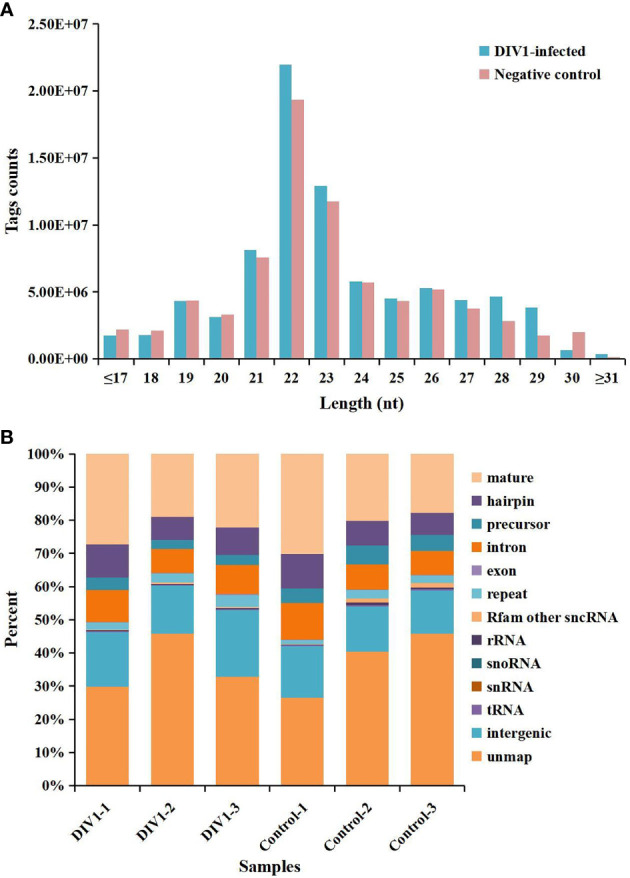
Length distribution, abundance, and composition of small RNA libraries of DIV1-infected and non-infected *Marsupenaeus japonicus* intestine. **(A)** Length distribution and abundance of small RNAs from the intestine of the DIV1-infected group and negative control group. The x-axis indicates the length of small RNAs, and the y-axis indicates the tag counts. **(B)** Composition of RNAs in each small RNA library. The x-axis indicates the different samples, and the y-axis indicates the percentage of RNAs. DIV1, decapod iridescent virus 1.

In order to identify miRNA expression profiles in the *M. japonicus* intestine under DIV1 infection, six small RNA libraries representing DIV1-infected and non-infected *M. japonicus* were constructed with total RNA, and then Illumina sequencing technology was used for small RNA sequencing. After quality filtering, a total of 74,190,726 and 81,759,203 clean tags were respectively obtained for the negative control group and DIV1-infected group (**Table 3**). The length distribution of clean tags was determined. The results showed that small RNAs of 22 nt in length were the most common, followed by those of 21 nt (**Figure 5A**). As shown in **Figure 5B**, all clean tags were annotated and classified into different categories, including rRNA, tRNA, snRNA, snoRNA, and miRNA. After other classes of small RNAs (rRNA, tRNA, snRNA, snoRNA, etc.) were removed, a total of 134 known miRNAs and 98 novel miRNAs were identified from the deep sequencing data using miRDeep2 software.

**Table 3:** Summary of miRNA-seq data.

Samples	Raw Tags Count	Clean Tags Count	Q20 (%)	Clean Tags Ratio (%)
DIV1-1	28,881,041	27,192,640	98.00	94.15
DIV1-2	27,944,852	26,544,371	97.70	94.99
DIV1-3	29,744,908	28,022,192	98.30	94.21
Control-1	21,529,313	20,219,118	98.00	93.91
Control-2	28,632,772	26,716,074	97.90	93.31
Control-3	28,918,111	27,255,534	97.80	94.25
Total	165,650,997	155,949,929	97.95	94.14

Sequencing reads were deposited into the SRA of the NCBI and are available with the accession number PRJNA752616 (https://www.ncbi.nlm.nih.gov/sra).

#### 3.2.2 Identification and Functional Characterization of Differentially Expressed MiRNAs

To characterize the host miRNAs involved in virus infection, the expression profiles of known and novel miRNAs in the DIV1-infected group and negative control group were compared. The miRNAs with *Q*-value < 0.05 and |log_2_(fold change)| > 0.1 were identified as DEMs. As shown in **Table 4**, a total of 17 known and 15 novel DEMs were identified between the DIV1-infected group and the negative control group.

**Table 4:** Identification miRNAs and their sequences.

MiRNA Name	Sequence (5′ –3′)	Length (nt)	*Q*-value	log2FC
miR-10	ACCCTGTAGATCCGAATTTGT	21	7.45E−03	1.37
miR-133-3p_2	TTGGTCCCCTTCAACCAGCTG	21	5.31E−03	−0.71
miR-193-3p_6	TACTGGCCTGCTAAGTCCCAA	21	3.56E−03	3.24
miR-210_4	TTGTGCGTGTGACAGCGGCT	20	2.05E−02	−0.89
miR-252_3	CTAAGTAGTAGTGCCGCAGGT	21	1.32E−07	7.25
miR-252b_1	CTAAGTAGTAGTGCCGCAGGTAA	23	4.75E−06	6.59
miR-263a-5p_1	AATGGCACTGGAAGAATTCACGG	23	8.53E−04	4.45
miR-263a-5p_2	AATGGCACTGGAAGAATTCAC	21	2.62E−02	3.33
miR-263b	CTTGGCACTGGAAGAATTCACAGA	24	6.90E−05	4.90
miR-263b_3	CTTGGCACTGGAAGAATTCACA	22	5.10E−04	4.70
miR-263b-5p_2	CTTGGCACTGGAAGAATTCAC	21	5.92E−04	4.85
miR-2b_4	TATCACAGCCACCTTTGATGAGC	23	5.31E−03	−0.48
miR-6489-3p	CGACGGAAAGGTGTCCAAGCTGG	23	8.59E−03	2.27
miR-6493-5p	ACGTCCGGCAGGTTTTACCCCT	22	2.13E−04	2.21
miR-750_3	CAGATCTAACTCTTCCAGCTCA	22	3.56E−03	−3.29
miR-750-3p_3	CCAGATCTAACTCTTCCAGCTC	22	2.67E−02	−0.49
miR-79-3p_1	TAAAGCTAGATTACCAAAGCA	21	1.18E−02	0.18
novel_mir24	TTGTGACCGTTATAATGGGC	20	5.81E−05	5.84
novel_mir31	TAGCACCATGTGAATTCAGTAC	22	2.13E−02	2.69
novel_mir34	TTCGTTGTCGTCGAAACCTGCA	22	5.10E−04	4.91
novel_mir40	ACGCCTTTGGTTTTACGGTCTTCG	24	4.53E−02	−1.20
novel_mir56	GGCGGGGGCTGGCGGCGCCGC	21	5.92E−04	4.21
novel_mir57	ATTAGGTTACCGCCGACC	18	3.63E−02	−4.56
novel_mir58	TCTCTGCGGCTCTTGGCTCACG	22	3.13E−02	−1.50
novel_mir60	TCTCCAGTAGCCTGTTAGGCAT	22	3.73E−04	−0.43
novel_mir63	CGCGTGGGGGATGACGGG	18	4.21E−02	−4.45
novel_mir7	TTTGGCAGTCGAGTAACTACA	21	2.39E−03	−0.26
novel_mir73	GTGGATTCTCTCCCGTTTTC	20	1.99E−07	7.53
novel_mir82	GCGCGGCCGGATGGTGGTG	19	4.21E−02	−4.44
novel_mir85	CGTGTTTATATTGTGGGTTTTC	22	2.13E−04	−0.76
novel_mir86	GCGCGCGGGCGGCGGTGGCTGC	22	1.96E−10	2.50
novel_mir9	ATTATCATTCTTTGGCGTCCGG	22	4.75E−06	1.10

Identification of the target mRNA of each DEM could provide clues as to the roles of miRNAs in the shrimp intestine response to DIV1 infection. The target genes of DEMs were predicted using RNAhybrid and miRanda. A total of 36,679 target genes were predicted for the DEMs using the two prediction programs. The target genes of DEMs were further processed for sequence annotation using the GO and KEGG databases.

**Figure 6:**
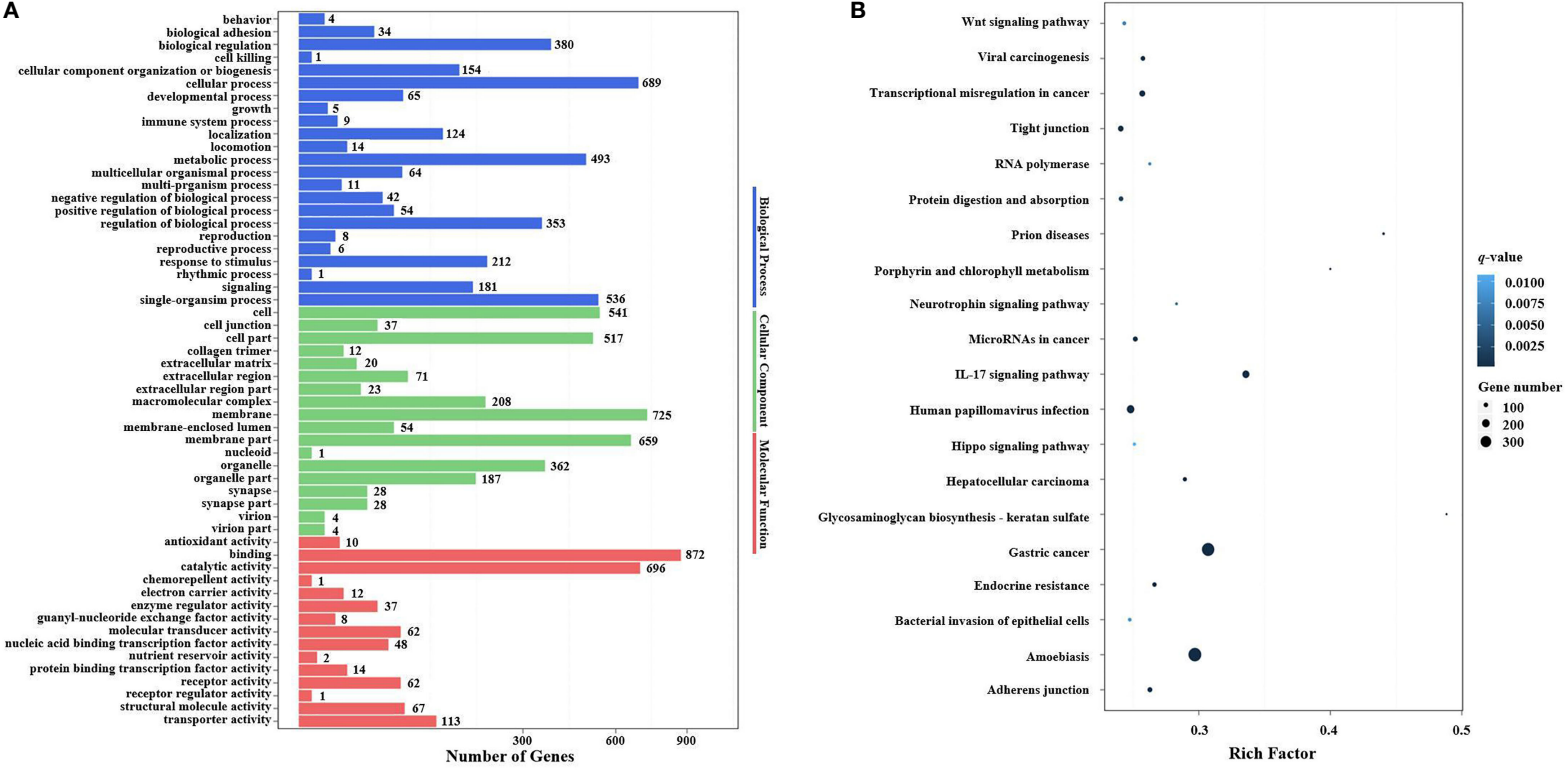
GO terms and KEGG pathway enrichment analyses of target genes of DEMs. **(A)** GO term enrichment analysis of target genes of DEMs. Three main GO categories: biological process, cellular component, and molecular function. The x-axis indicates the number of genes, and the y-axis indicates the GO terms (level 2). **(B)** Top 20 KEGG pathways enriched in target genes of DEMs (level 3). The x-axis indicates the ratio of the number of genes in the DEGs, and the y-axis indicates the pathways. GO, Gene Ontology; KEGG, Kyoto Encyclopedia of Genes and Genomes; DEMs, differentially expressed miRNAs; DEGs, differentially expressed genes.

In the GO enrichment analysis, the predicted target genes were clustered into three GO categories, biological process, cellular component, and molecular function. These three main GO categories were further classified into 56 subcategories (level 2). In the biological process category, 689 and 536 target genes of DEMs were enriched in “cellular processes” and “single biological processes,” respectively. In the cellular component category, 725 and 659 target genes of DEMs were enriched in “cell processes” and “single biological processes,” respectively. For the molecular function category, 872 and 696 target genes of DEMs were enriched in “binding” and “catalytic activity,” respectively (**Figure 6A**).

In the KEGG pathway enrichment analysis, a total of 3,915 target genes of DEMs were enriched in 324 KEGG pathways (level 3). The top 20 KEGG pathways influenced by DIV1 infection are shown in **Figure 6B**. These pathways contained three common immune-related pathways, including the Wnt signaling pathway (82 target genes of DEMs), IL-17 signaling pathway (182 target genes of DEMs), and Hippo signaling pathway (65 target genes of DEMs). Several pathways related to the intestinal barrier function were also significantly enriched, including Tight junction (135 target genes of DEMs), Adherens junction (113 target genes of DEMs), and Bacterial invasion of epithelial cells (73 target genes of DEMs). It is worth noting that Glycosaminoglycan biosynthesis—keratan sulfate was also significantly enriched in the KEGG pathway enrichment analysis of target genes of DEMs.

### 3.3 qRT-PCR Validation of Differentially Expressed Genes and Differentially Expressed MiRNAs

**Figure 7:**
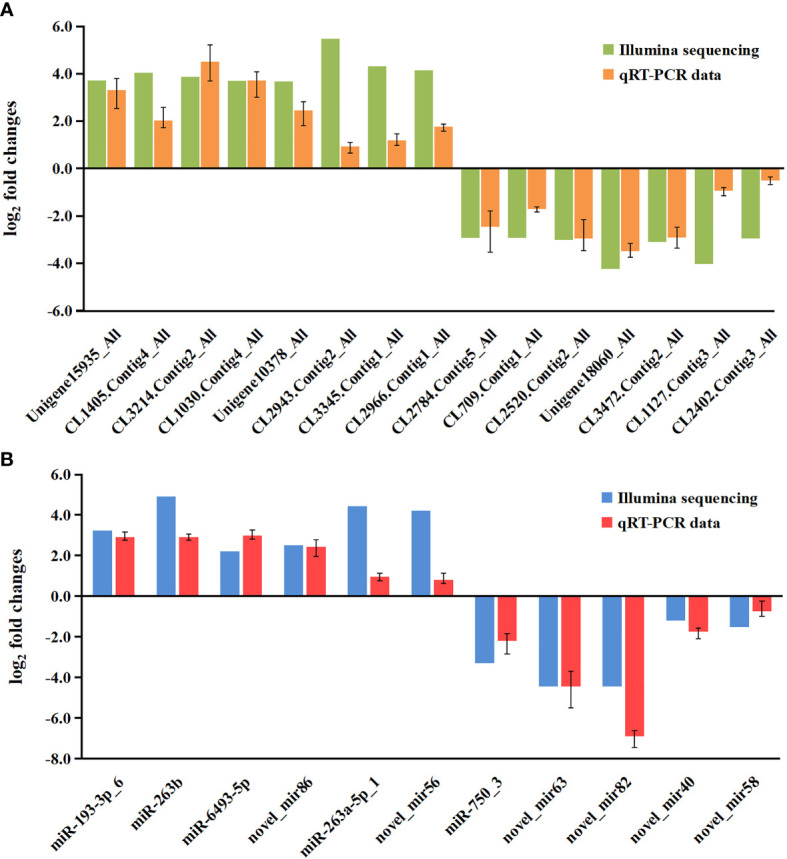
Validation of RNA-seq results *via* qRT-PCR. **(A)** Comparison of the expression profiles of 10 DEGs as determined by Illumina sequencing and qRT-PCR. The x-axis indicates the names of the DEGs, and the y-axis represents the log_2_(fold change). **(B)** Comparison of the expression profiles of 8 DEMs as determined by Illumina sequencing and qRT-PCR. The x-axis indicates the names of the DEMs, and the y-axis represents the log_2_(fold change). DEGs, differentially expressed genes.

To validate the mRNA and miRNA expression profiles from the Illumina sequencing results, a total of 15 DEGs and 11 DEMs were randomly selected for qRT-PCR, including eight upregulated DEGs (Unigene15935_All, CL1405.Contig4_All, CL3214.Contig2_All, CL1030.Contig4_All, Unigene10378_All, CL2943.Contig2_All, CL3345.Contig1_All, and CL2966.Contig1_All), seven downregulated DEGs (CL2784.Contig5_All, CL709.Contig1_All, CL2520.Contig2_All, Unigene18060_All, CL3472.Contig2_All, CL1127.Contig3_All, and CL2402.Contig3_All), six upregulated DEMs (miR-193-3p_6, miR-263b, miR-6493-5p, novel_mir86, miR-263a-5p_1, and novel_mir56), and five downregulated DEMs (miR-750_3, novel_mir63, novel_mir82, novel_mir40, and novel_mir58). As shown in **Figures 7A, B**, the expression patterns of these tested genes were consistent across both methods. This result demonstrates that the mRNA and miRNA expression profiles derived from RNA-seq were reliable and confirm the expression changes of these genes in response to DIV1 infection.

### 3.4 Correlation Analysis Between Differentially Expressed MiRNAs and Differentially Expressed Genes in the Intestine of *Marsupenaeus japonicus* Under Decapod Iridescent Virus 1 Infection

**Figure 8:**
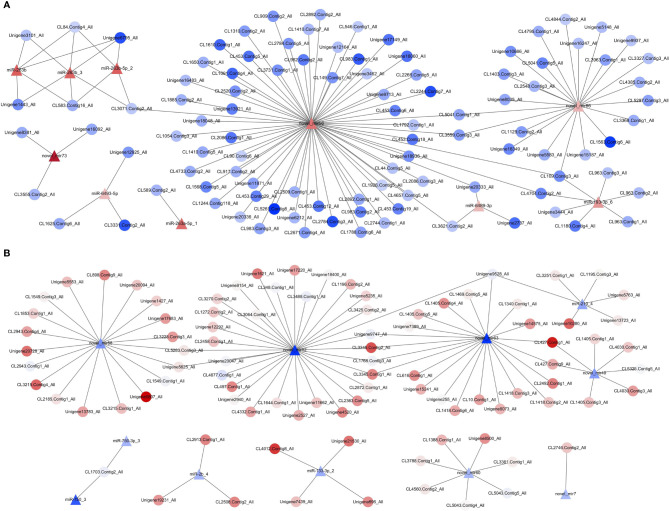
Network analysis for miRNA/mRNA interaction. **(A)** MiRNA–mRNA network was formed by 10 upregulated DEMs and 119 downregulated DEGs. **(B)** MiRNA–mRNA network was formed by 11 downregulated DEMs and 103 upregulated DEGs. Red indicates that genes (miRNAs and mRNAs) were upregulated, and blue represents that genes (miRNAs and mRNAs) were downregulated; the darker the color, the greater the fold change. △ represents miRNA, and ○ represents mRNA. DEGs, differentially expressed genes; DEMs, differentially expressed miRNAs.

To further understand the functional relationship between DEMs and DEGs, correlations between DEMs and DEGs were evaluated. In total, 21 DEMs were negatively correlated with 194 DEGs, from a total of 223 correlations. Two miRNA–mRNA networks were found. One miRNA–mRNA network was formed by 10 upregulated DEMs and 119 downregulated DEGs (**Figure 8A**), and the other miRNA–mRNA network was formed by 11 downregulated DEMs and 103 upregulated DEGs (**Figure 8B**). The results showed that one miRNA can regulate multiple mRNAs, and one mRNA can also be regulated by multiple miRNAs, thus forming a complex miRNA–mRNA regulatory network.

### 3.5 Kyoto Encyclopedia of Genes and Genomes and Gene Ontology Analysis of Target Differentially Expressed Genes of Differentially Expressed MiRNAs

**Figure 9:**
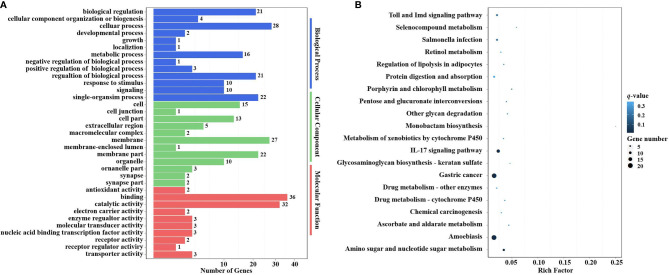
GO terms and KEGG pathway enrichment analyses of target DEGs of DEMs. **(A)** GO term enrichment analysis of target DEGs of DEMs. Three main GO categories: biological process, cellular component, and molecular function. The x-axis indicates the number of genes, and the y-axis indicates the GO terms (level 2). **(B)** Top 20 KEGG pathway enrichment of DEG genes of DEMs (level 3). The x-axis indicates the ratio of the number of genes in the DEGs, and the y-axis indicates the pathways. GO, Gene Ontology; KEGG, Kyoto Encyclopedia of Genes and Genomes; DEGs, differentially expressed genes; DEMs, differentially expressed miRNAs.

In order to investigate the possible role of miRNAs in the immune response of shrimp against DIV1 infection, 223 target DEGs of DEMs were analyzed using the GO and KEGG databases to study the miRNA functions. In the GO enrichment analysis, the target DEGs of DEMs were mainly enriched in three GO categories with 35 subcategories (level 2). In the biological process category, 28 and 22 target DEGs of DEMs were enriched in “cellular processes” and “single biological processes,” respectively. In the cellular component category, 27 and 22 target DEGs of DEMs were enriched in “membrane” and “membrane part,” respectively. For the molecular function category, 36 and 32 target genes of DEMs were enriched in “binding” and “catalytic activity,” respectively (**Figure 9A**).

In the KEGG pathway enrichment analysis, a total of 115 target DEGs of DEMs were enriched in 42 KEGG pathways (level 3). The top 20 enriched KEGG pathways are shown in **Figure 9B**. Two well-known immune-related pathways were identified through KEGG pathway enrichment analysis, i.e., the Toll and IMD signaling pathways (7 target DEGs of DEMs) and the IL-17 signaling pathway (13 target DEGs of DEMs). Moreover, two vitamin metabolism-related pathways were also identified, i.e., Retinol metabolism (3 target DEGs of DEMs) and Ascorbate and aldarate metabolism (3 target DEGs of DEMs). In addition, several other metabolic pathways were also significantly enriched, including Selenocompound metabolism (2 target DEGs of DEMs), Porphyrin and chlorophyll metabolism (3 target DEGs of DEMs), Metabolism of xenobiotics by cytochrome P450 (3 target DEGs of DEMs), Drug metabolism—other enzymes (4 target DEGs of DEMs), Drug metabolism—cytochrome P450 (3 target DEGs of DEMs), and Amino sugar and nucleotide sugar metabolism (8 target DEGs of DEMs). It is worth noting that Glycosaminoglycan biosynthesis—keratan sulfate (2 target DEGs of DEMs) was also significantly enriched in the KEGG pathway enrichment analysis.

**Figure 10:**
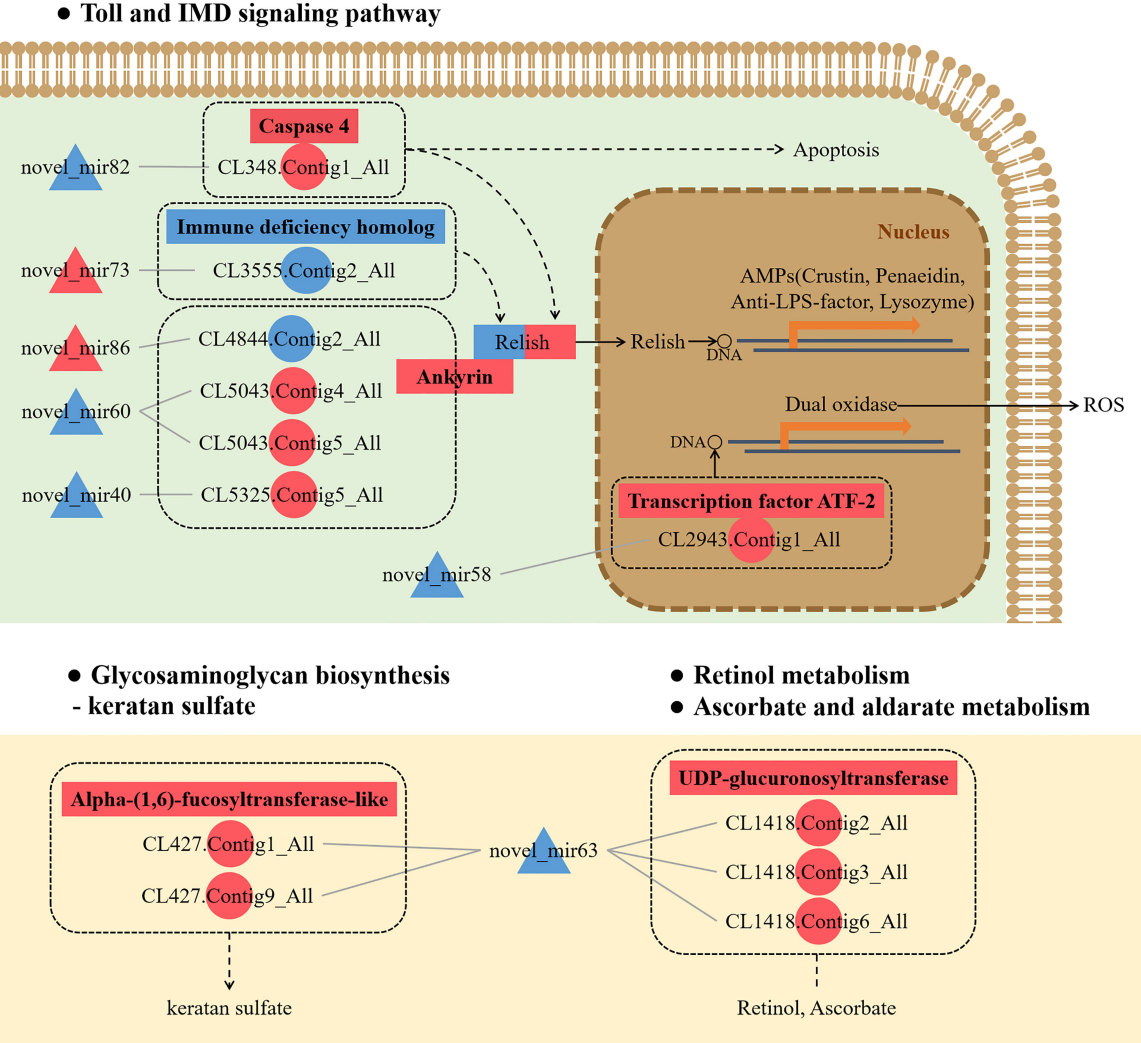
Conceptual diagram of miRNA/mRNA pairs in four potential immune pathways, including the Toll and IMD signaling pathways, Glycosaminoglycan biosynthesis—keratan sulfate, Retinol metabolism, and Ascorbate and aldarate metabolism. Intestinal miRNA can affect the expression of mRNA, thereby regulating apoptosis, AMPs, ROS, keratan sulfate biosynthesis, and VA and VC metabolism, thus resisting DIV1 infection (see the text for detail). Red indicates that genes (miRNAs and mRNAs) were upregulated, and blue indicates that genes (miRNAs and mRNAs) were downregulated. △ represents miRNA, and ○ represents mRNA. AMP, antimicrobial peptide; ROS, reactive oxygen species; VA, vitamin A; VC, vitamin C; DIV1, decapod iridescent virus 1.

To further understand the role of miRNA/mRNA pairs in anti-DIV1 infection, an in-depth analysis of the four significantly enriched potential immune pathways (Toll and IMD signaling pathways, Glycosaminoglycan biosynthesis—keratan sulfate, Retinol metabolism, and Ascorbate and aldarate metabolism) was performed (**Figure 10**). The results showed that in the Toll and IMD signaling pathways, the downregulation of novel_mir82 resulted in the upregulation of its target gene caspase 4, which promotes apoptosis and the expression of Relish. Novel_mir73, novel_mir86, novel_mir60, and novel_mir40 can affect the translocation of Relish to the nucleus by regulating the expression of IMD and Ankyrin, thereby regulating the synthesis of downstream AMPs. The downregulation of novel_mir58 led to the upregulated expression of its target gene transcription factor ATF-2 (ATF-2), which promotes dual oxidase (DUOX) to produce reactive oxygen species (ROS). In the Glycosaminoglycan biosynthesis—keratan sulfate pathway, novel_mir63 regulated the expression of alpha-(1,6)-fucosyltransferase-like (α1-6FucT) to affect the biosynthesis of chondroitin sulfate. It is worth noting that in the Retinol metabolism and Ascorbate and aldarate metabolism pathways, novel_mir63 can also regulate the metabolism of retinol and ascorbate by affecting UDP-glucuronosyltransferase (UGT). All miRNA–mRNA pairs that are potentially involved in the immune response against DIV1 infection are listed in **Table 5**.

**Table 5:** Candidate miRNA–mRNA pairs involved in *Marsupenaeus japonicus* intestinal immune response against DIV1.

DEM name	Target DEG Name	Homolog Function	Species	MiRNAlog_2_FC	Targetlog_2_FC
***Toll and IMD signaling pathways***					
novel_mir40	CL5325.Contig5_All	Ankyrin-1-like	*M. japonicus*	−1.20	1.49
novel_mir58	CL2943.Contig1_All	Transcription factor ATF-2	*Litopenaeus vannamei*	−1.50	1.34
novel_mir60	CL5043.Contig4_All	Ankyrin-1-like	*L. vannamei*	−0.43	1.61
novel_mir60	CL5043.Contig5_All	Serine/threonine-protein phosphatase 6 regulatory ankyrin repeat subunit A-like	*Amphimedon queenslandica*	−0.43	1.30
novel_mir73	CL3555.Contig2_All	Immune deficiency homolog	*M. japonicus*	7.53	−1.81
novel_mir82	CL348.Contig1_All	Caspase 4	*L. vannamei*	−4.44	1.43
novel_mir86	CL4844.Contig2_All	Serine/threonine-protein phosphatase 6 regulatory ankyrin repeat subunit A-like isoform X1	*L. vannamei*	2.50	−1.10
***IL-17 signaling pathway***					
miR-6493-5p	Unigene12925_All	Mucin-5AC-like isoform X2	*Habropoda laboriosa*	2.21	−2.86
miR-750_3	CL1703.Contig2_All	Uncharacterized protein LOC113809504	*L. vannamei*	−3.29	1.24
novel_mir56	CL2784.Contig3_All	Uncharacterized protein LOC113823076	*L. vannamei*	4.21	−5.43
novel_mir56	CL2784.Contig5_All	Uncharacterized protein LOC113823075 isoform X3	*L. vannamei*	4.21	−2.93
novel_mir56	CL917.Contig2_All	UNC93-like protein MFSD11	*L. vannamei*	4.21	−1.89
novel_mir56	Unigene3462_All	Uncharacterized protein LOC113822805	*L. vannamei*	4.21	−1.18
novel_mir58	Unigene20728_All	Hypothetical protein C7M84_008404	*L. vannamei*	−1.50	4.81
novel_mir60	CL1388.Contig1_All	Hypothetical protein C7M84_016913	*L. vannamei*	−0.43	3.13
novel_mir63	CL1768.Contig3_All	Mucin-5AC-like isoform X2	*H. laboriosa*	−4.45	1.39
novel_mir63	CL616.Contig1_All	Uncharacterized protein LOC113812248	*L. vannamei*	−4.45	4.15
novel_mir82	CL2872.Contig1_All	Hypothetical protein C7M84_008734	*L. vannamei*	−4.44	1.90
novel_mir82	CL348.Contig1_All	Caspase 4	*L. vannamei*	−4.44	1.43
novel_mir86	CL5297.Contig3_All	Phospholipase D gamma 3-like	*Hyalella azteca*	2.50	−2.50
***Complement and coagulation cascades***					
novel_mir63	CL3345.Contig1_All	Mite allergen Der p 3-like	*L. vannamei*	−4.45	4.32
novel_mir63	CL3345.Contig2_All	Mite allergen Der p 3-like	*L. vannamei*	−4.45	5.60
***Peroxisome***					
miR-210_4	Unigene10280_All	Masquerade-like protein, partial	*L. vannamei*	−0.89	5.15
novel_mir82	Unigene18400_All	Copper/zinc superoxide dismutase isoform 5	*M. japonicus*	−4.44	1.64
novel_mir82	Unigene5235_All	Indole-3-acetaldehyde oxidase-like	*L. vannamei*	−4.44	2.44
***NOD-like receptor signaling pathway***					
novel_mir58	CL1549.Contig1_All	Uncharacterized protein LOC113821210	*L. vannamei*	−1.50	1.37
novel_mir58	CL1549.Contig3_All	Uncharacterized protein LOC113821210	*L. vannamei*	−1.50	1.75
novel_mir82	CL348.Contig1_All	Caspase 4	*L. vannamei*	−4.44	1.43
***PI3K-Akt signaling pathway***					
novel_mir56	CL1568.Contig5_All	Protein draper-like	*L. vannamei*	4.21	−3.48
novel_mir58	CL2943.Contig1_All	Transcription factor ATF-2	*L. vannamei*	−1.50	1.34
novel_mir58	CL898.Contig9_All	Dentin sialophosphoprotein-like isoform X3	*L. vannamei*	−1.50	4.30
novel_mir86	CL3368.Contig1_All	Vascular endothelial growth factor receptor precursor	*L. vannamei*	2.50	−2.11
novel_mir86	CL3963.Contig1_All	Collagen alpha-1(I) chain-like isoform X2	*H. azteca*	2.50	−2.55
***MAPK signaling pathway***					
novel_mir58	CL1853.Contig1_All	Map kinase-interacting serine/threonine, partial	*Procambarus clarkii*	−1.50	2.46
novel_mir58	CL2943.Contig1_All	Transcription factor ATF-2	*L. vannamei*	−1.50	1.34
novel_mir86	CL3368.Contig1_All	Vascular endothelial growth factor receptor precursor	*L. vannamei*	2.50	−2.11
***Lysosome***					
miR-133-3p_2	Unigene896_All	Glucosylceramidase-like	*L. vannamei*	−0.71	4.21
novel_mir82	CL1196.Contig2_All	Beta-hexosaminidase subunit alpha-like	*L. vannamei*	−4.44	3.11
***Necroptosis***					
novel_mir63	CL1469.Contig5_All	Von Willebrand factor A domain-containing protein 5A-like	*L. vannamei*	−4.45	1.85
novel_mir82	CL348.Contig1_All	Caspase 4	*L. vannamei*	−4.44	1.43
***Apoptosis***					
novel_mir63	CL1469.Contig5_All	Von Willebrand factor A domain-containing protein 5A-like	*L. vannamei*	−4.45	1.85
novel_mir82	CL348.Contig1_All	Caspase 4	*L. vannamei*	−4.44	1.43
***p53 signaling pathway***					
novel_mir82	CL348.Contig1_All	Caspase 4	*L. vannamei*	−4.44	1.43
***Bacterial invasion of epithelial cells***					
novel_mir40	CL1405.Contig3_All	Leucine-rich repeat extensin-like protein 3	*L. vannamei*	−1.20	2.85
novel_mir63	CL1405.Contig4_All	Leucine-rich repeat extensin-like protein 3	*L. vannamei*	−4.45	4.03
novel_mir56	CL1610.Contig1_All	Paxillin-like	*L. vannamei*	4.21	−4.61
novel_mir82	CL3064.Contig1_All	Basic proline-rich protein-like, partial	*Canis lupus dingo*	−4.44	1.70
***Adherens junction***					
novel_mir40	CL1405.Contig3_All	Leucine-rich repeat extensin-like protein 3	*L. vannamei*	−1.20	2.85
novel_mir56	CL4657.Contig5_All	Receptor-type tyrosine-protein phosphatase delta-like	*L. vannamei*	4.21	−1.28
novel_mir63	CL1405.Contig4_All	Leucine-rich repeat extensin-like protein 3	*L. vannamei*	−4.45	4.03
novel_mir82	CL3064.Contig1_All	Basic proline-rich protein-like, partial	*C. lupus dingo*	−4.44	1.70
***C-type lectin receptor signaling pathway***					
novel_mir82	CL348.Contig1_All	Caspase 4	*L. vannamei*	−4.44	1.43
***Retinol metabolism***					
novel_mir63	CL1418.Contig2_All	UDP-glucuronosyltransferase	*L. vannamei*	−4.45	2.75
novel_mir63	CL1418.Contig3_All	UDP-glucuronosyltransferase	*L. vannamei*	−4.45	3.05
novel_mir63	CL1418.Contig6_All	UDP-glucuronosyltransferase	*L. vannamei*	−4.45	3.41
***Ascorbate and aldarate metabolism***					
novel_mir63	CL1418.Contig2_All	UDP-glucuronosyltransferase	*L. vannamei*	−4.45	2.75
novel_mir63	CL1418.Contig3_All	UDP-glucuronosyltransferase	*L. vannamei*	−4.45	3.05
novel_mir63	CL1418.Contig6_All	UDP-glucuronosyltransferase	*L. vannamei*	−4.45	3.41
***Glycosaminoglycan biosynthesis—keratan sulfate***					
novel_mir63	CL427.Contig1_All	Alpha-(1,6)-fucosyltransferase-like	*L. vannamei*	−4.45	6.87
novel_mir63	CL427.Contig9_All	Alpha-(1,6)-fucosyltransferase-like	*L. vannamei*	−4.45	3.37
***Glycosaminoglycan biosynthesis—chondroitin sulfate/dermatan sulfate***					
novel_mir82	Unigene17220_All	Hypothetical protein C7M84_016157	*L. vannamei*	−4.44	3.71
***Glycosaminoglycan biosynthesis—heparan sulfate/heparin***					
novel_mir82	Unigene17220_All	Hypothetical protein C7M84_016157	*L. vannamei*	−4.44	3.71
***Glycosphingolipid biosynthesis—globo and isoglobo series***					
novel_mir82	CL1196.Contig2_All	Beta-hexosaminidase subunit alpha-like	*L. vannamei*	−4.44	3.11
***Glycosphingolipid biosynthesis—ganglio series***					
novel_mir82	CL1196.Contig2_All	Beta-hexosaminidase subunit alpha-like	*L. vannamei*	−4.44	3.11

DIV1, decapod iridescent virus 1.

## 4 Discussion

With the development of sequencing technology, miRNA-seq and mRNA-seq analysis technologies have been widely applied in the study of human and animal disease diagnosis and pathogenesis. At present, our understanding of the role of miRNA in the shrimp intestinal antiviral immune response is very limited. In this study, we firstly reported both intestinal miRNA-seq and mRNA-seq analyses of *M. japonicus* infected with DIV1. The integrated analysis revealed the process of DIV1 invasion and the mechanism of the shrimp intestinal antiviral immune response.

Through the mRNA-seq analysis, a total of 54,107 unigenes were *de novo* assembled, and 1,234 upregulated genes and 742 downregulated genes were screened based on a *Q*-value < 0.05 and |log_2_(fold change)| > 1 cutoff. All DEGs were mapped in the GO and KEGG databases. In the GO enrichment analysis, the GO term triose-phosphate isomerase activity was promoted and significantly enriched in *M. japonicus* under DIV1 infection. TPI, which can catalyze the interconversion of glyceraldehyde-3-phosphate (GAP) and dihydroxyacetone phosphate (DHAP), plays a vital role in both glycolysis and phospholipid biosynthesis. Previous studies have shown that WSSV and DIV1 infection can cause significant changes in several metabolic processes involved in TPI, including glycolysis, thereby promoting the synthesis of a large number of phospholipids to aid virus replication (13, 55). Among the top 20 KEGG pathways, three marker pathways of the Warburg effect were significantly enriched, including Pyruvate metabolism, Glycolysis/Gluconeogenesis, and TCA cycle. The Warburg effect was first found in cancer cells, and it was later found that certain vertebrate and invertebrate viruses can also induce the Warburg effect, such as human papillomavirus (HPV) and WSSV (56–58). The Warburg effect can not only weaken aerobic respiration under aerobic conditions to help cancer cells or viruses escape apoptosis and the host immune response but also perform efficient glycolysis to obtain sufficient energy and substances to promote the proliferation of cancer cells or the replication of viruses (59, 60). In the current study, the triose-phosphate isomerase activity GO terms and the Warburg effect marker pathways in DIV1-infected *M. japonicus* were significantly enriched, which means that DIV1 may promote its own replication by regulating the host’s intestinal metabolism.

Retinol, also known as vitamin A (VA), is a micronutrient necessary for maintaining mucosal epithelial cell renewal and damage repair; it plays an important and active role in regulating intestinal mucosal immunity (61). Several studies have shown that dietary VA can improve the lysozyme activity of serum and the phagocytic ability of hemocytes in aquatic organisms (62–65). Ascorbic acid, also known as vitamin C (VC), has a variety of physiological functions, including growth promotion, wound repair, immune enzyme activity improvement, and anti-stress (66). Many studies have shown that dietary VC can promote the recovery of shrimp after molting, increase the weight gain rate, and improve the disease resistance and the survival rate of shrimp (67–70). In this study, the Retinol metabolism and Ascorbate and aldarate metabolism pathways were significantly changed after DIV1 infection, indicating that DIV1 can affect intestinal mucosal immunity and cellular immunity of *M. japonicus* by affecting the metabolism of VA and VC.

Glycosphingolipids, as important components of eukaryotic cell membranes, are widely involved in various physiological activities, including signal transduction, immunity, apoptosis, and pathogen invasion (71). Based on their core structures, glycosphingolipids are mainly divided into four series, including ganglio-, globo/isoglobo-, lacto/neolacto, and gala/neogala series. In recent years, many studies have found that glycosphingolipids act as cofactors to participate in the adsorption, entry, and replication stages of a variety of viruses, including HIV, Norovirus (NoVs), Dengue virus (DENV1), and Coccolithoviruses (EhVs) (72–76). Similarly, in this study, glycosphingolipid biosynthesis-related pathways were activated and significantly enriched after DIV1 infection, which implies that DIV1 may promote its own invasion and replication by mediating the host’s glycosphingolipid biosynthesis.

Chondroitin sulfate, dermatan sulfate, and keratan sulfate are three types of sulfated glycosaminoglycans; they are important parts of animal connective tissues and have various biological functions such as anticoagulation and antiviral (77). In recent years, sulfated glycosaminoglycans have been found to have great antiviral potential. Several studies have shown that sulfated glycosaminoglycans (such as chondroitin sulfate and sea cucumber sulfated polysaccharide) exhibit significant inhibitory activity against a variety of viruses *in vitro*, including HIV, severe acute respiratory syndrome coronavirus 2 (SARS-CoV-2), and Grass carp reovirus (GCRV) type I and type III (78–80). However, there is still no research on the inhibitory effect of glycosaminoglycans such as chondroitin sulfate on shrimp viruses. In this study, DIV1 infection caused significant enrichment of glycosaminoglycan biosynthesis-related pathways in the intestine of *M. japonicus*. This phenomenon implies that glycosaminoglycans, such as chondroitin sulfate and keratan sulfate, play important roles in resisting DIV1 infection. The specific mechanism requires further study.

Clearly, under DIV1 infection, the intestinal transcriptome results are very different from the previous hemocyte and hepatopancreas transcriptome results. In the intestinal transcriptome, more metabolic-related pathways were significantly enriched, which reveals the metabolic changes after shrimp infection with DIV1. These metabolic-related pathways play important roles in virus infection and host immunity and cannot be ignored.

The miRNA-seq analysis identified a total of 134 known miRNAs and 98 novel miRNAs from the deep sequencing data, and 19 upregulated miRNAs and 13 downregulated miRNAs were screened with a cutoff of *Q*-value < 0.05 and |log_2_(fold change)| > 0.1. To understand the role of shrimp intestinal miRNAs under DIV1 infection, the target genes of DEMs were predicted. In the KEGG pathway enrichment analysis of target genes of DEMs, three common immune-related pathways were significantly enriched, including the Wnt signaling pathway, IL-17 signaling pathway, and Hippo signaling pathway. The Wnt signaling pathway refers to a set of evolutionarily conserved signaling pathways that are involved in a variety of important biological processes, including cell proliferation and differentiation, intestinal development, and tissue homeostasis (81–83). Though there has been little research on the Wnt signaling pathway in crustaceans, a recent study found that Wnt genes might participate in the immune response of *L. vannamei* after infection with *Staphylococcus aureus*, *Vibrio parahaemolyticus*, and WSSV (84). The Hippo signaling pathway, originally considered to be a growth regulation pathway, has recently been found to play indispensable roles in the regulation of innate anti-bacterial immunity and anti-viral immunity (85, 86). Several studies have demonstrated that both the Wnt signaling pathway and Hippo signaling pathway are involved in intestinal regeneration and intestinal homeostasis (87–90). These results imply that shrimp intestinal miRNAs participate in the intestinal immune response against DIV1 infection by regulating genes in the immune signaling pathway.

It is worth noting that three pathways related to the barrier function of the intestine were significantly enriched, including Tight junction, Adherens junction, and Bacterial invasion of epithelial cells. This indicates that DIV1 infection can destroy the mechanical barrier of the intestinal mucosa by weakening the tight junction and adherens junction of intestinal mucosal epithelial cells, eventually causing bacteria to infect the host through the intestine. MiRNAs might play important roles in maintaining the stability of the intestinal mucosal mechanical barrier. In addition, similar to the KEGG pathway enrichment analysis of DEGs, the Glycosaminoglycan biosynthesis—keratan sulfate pathway was significantly enriched in the KEGG pathway enrichment analysis of the target genes of DEMs. This indicates that miRNAs play a role in the immune response against DIV1 infection by regulating the biosynthesis of glycosaminoglycans.

Following the mRNA-seq and miRNA-seq analyses, correlation analysis of DEMs and DEGs in the intestine was carried out to further explore the regulatory function of differentially expressed miRNA/mRNA pairs in the process of DIV1 infection. A total of 223 correlations were performed and analyzed for enrichment in the GO and KEGG databases. In the functional annotation analysis of DEMs/DEGs pairs, the Toll and IMD signaling pathways were found to be significantly enriched after DIV1 infection. Further analysis revealed that five DEMs were involved in the regulation of the Toll and IMD signaling pathways by affecting the expression of caspase 4, ATF2, IMD, and Ankyrin. The Toll and IMD signaling pathways are two NF-κB signaling pathways that are considered to be the main pathways involved in the regulation of the immune response in invertebrates (91, 92). Many studies have demonstrated that the Toll and IMD signaling pathways can inhibit viral and bacterial infections by regulating the expression of AMPs (such as crustin (93, 94), penaeidin (95, 96), anti-lipopolysaccharide (anti-LPS) factor (97–99), and lysozyme (100, 101), promoting apoptosis (102–104) and producing ROS (105). This indicates that miRNA/mRNA pairs play an important role in combating DIV1 infection and secondary bacterial infections caused by DIV1 infection. It is worth noting that in the correlation analysis of DEMs and DEGs, Glycosaminoglycan biosynthesis-related pathways and both VA and VC metabolism-related pathways were also significantly enriched, indicating that differentially expressed miRNA/mRNA pairs can regulate these pathways to resist DIV1 infection. Based on the above results, it is clear that intestinal miRNAs play important and unexpected roles in the process of viral infection and host immunity. The immune molecular mechanisms of these miRNAs require further study and verification.

## 5 Conclusions

In conclusion, through mRNA-seq and miRNA-seq analyses as well as correlation analysis between the two, the present study revealed that the intestine of *M. japonicus* can regulate glycosaminoglycan biosynthesis, vitamin metabolism, immune pathway activation, immunity enzyme activity promotion, AMP expression, ROS production, and cell apoptosis throug miRNAs to participate in the host’s antiviral immune response. Moreover, DIV1 can influence Warburg effect-related pathways, glycosphingolipid biosynthesis-related pathways, and the tight junction and adhesion junction of the intestinal mucosal epithelium through the host’s miRNAs and mRNA to promote its own invasion and replication. The addition of dietary glycosaminoglycans and vitamins may help enhance shrimp intestinal immunity and resist DIV1 infection.

## Data Availability Statement

The datasets presented in this study can be found in online repositories. The names of the repository/repositories and accession number(s) can be found below: https://www.ncbi.nlm.nih.gov/sra, accession ID: PRJNA720250

## Ethics Statement

The animal study was reviewed and approved by the ethics review board of the Institutional Animal Care and Use Committee in Guangdong Ocean University.

## Author Contributions

ZH, CS, and SZ contributed to the conception and design of the study. ZH, YZ, DH, XH, ZF, and LL collected the samples and performed the experiments. ZH wrote the first draft of the manuscript. SZ performed the writing review and editing. CS contributed to the project administration and funding acquisition. All authors contributed to manuscript revision, and read and approved the submitted version.

## Funding

This research was funded by the key research and development projects in Guangdong Province (Grant No. 2020B0202010009), the project of 2019 Annual Guangdong Provincial Special Financial Fund (Grant No. 231419025), and the Fangchenggang Science and Technology Plan Project (Grant No. AD19008017).

## Conflict of Interest

The authors declare that the research was conducted in the absence of any commercial or financial relationships that could be construed as a potential conflict of interest.

## Publisher’s Note

All claims expressed in this article are solely those of the authors and do not necessarily represent those of their affiliated organizations, or those of the publisher, the editors and the reviewers. Any product that may be evaluated in this article, or claim that may be made by its manufacturer, is not guaranteed or endorsed by the publisher.
